# The Default Mode Network Supports Episodic Memory in Cognitively Unimpaired Elderly Individuals: Different Contributions to Immediate Recall and Delayed Recall

**DOI:** 10.3389/fnagi.2018.00006

**Published:** 2018-01-24

**Authors:** Lijuan Huo, Rui Li, Pengyun Wang, Zhiwei Zheng, Juan Li

**Affiliations:** ^1^Key Laboratory of Mental Health, Center on Aging Psychology, Institute of Psychology, Chinese Academy of Sciences, Beijing, China; ^2^Department of Psychology, University of Chinese Academy of Sciences, Beijing, China

**Keywords:** immediate recall, delayed recall, default mode network, functional connectivity, regional homogeneity

## Abstract

While the neural correlates of age-related decline in episodic memory have been the subject of much interest, the spontaneous functional architecture of the brain for various memory processes in elderly adults, such as immediate recall (IR) and delayed recall (DR), remains unclear. The present study thus examined the neural correlates of age-related decline of various memory processes. A total of 66 cognitively normal older adults (aged 60–80 years) participated in this study. Memory processes were measured using the Auditory Verbal Learning Test as well as resting-state brain images, which were analyzed using both regional homogeneity (ReHo) and correlation-based functional connectivity (FC) approaches. We found that both IR and DR were significantly correlated with the ReHo of these critical regions, all within the default mode network (DMN), including the parahippocampal gyrus, posterior cingulate cortex/precuneus, inferior parietal lobule, and medial prefrontal cortex. In addition, DR was also related to the FC between these DMN regions. These results suggest that the DMN plays different roles in memory retrieval across different retention intervals, and connections between the DMN regions contribute to memory consolidation of past events in healthy older people.

## Introduction

Episodic memory refers to the human memory system that enables people to remember specific past events or information (Tulving et al., 1994). Its formation encompasses multiple processes, including encoding or the acquisition of learning material, consolidation or the retention of newly acquired information, and retrieval of the information already stored (Tromp et al., 2015). Aging is accompanied with episodic memory decline, which can lead to different degrees of damage in all associated memory processes (Koen and Yonelinas, 2014). It is therefore important to further investigate age-related decline of episodic memory and the underlying neural mechanisms of neurodegenerative diseases, and to use this information to formulate potential memory interventions.

Among the variety of standardized neuropsychological tests that measure episodic memory, list-learning tests are perhaps the most commonly used. Verbal list-learning tests are commendably employed to assess early cognitive decline in patients with mild cognitive impairment (MCI) and Alzheimer’s disease (AD), as well as to predict diagnostic conversion to AD (Arnaiz and Almkvist, 2003; Karrasch et al., 2005; Guo et al., 2009). These tests involve learning across multiple trials and typically produce several memory indices, such as immediate free recall, delayed free recall, and recognition (Ivnik et al., 1990; Bleecker et al., 2005). In terms of the different retention intervals, episodic memory is measured by considering immediate recall (IR) and delayed recall (DR), which require participants to retrieve memories immediately after learning and 30 min after learning, respectively.

Verbal learning and memory retrieval with different retention intervals may encompass different memory processes. For example, participants do not have enough time to rehearse or consolidate memory in the case of IR. However, during a delayed period in the case of DR, consolidation can occur; this stabilizes the newly encoded memory, and enhances and integrates the new information with pre-existing long-term memories (Marshall and Born, 2007). Memory consolidation relies on a dialog between the neocortex and hippocampus (Winocur et al., 2007). The newly acquired memory representations are stored in the hippocampus and are gradually redistributed and transferred to neocortical regions via the strengthening of cortico-cortical circuits (Rasch and Born, 2007; Takashima et al., 2009). Thus, we can speculate that DR depends on neocortex-subcortex (hippocampus) connections and cortico-cortical connections, while IR does not. Moreover, different memory deficits of IR and DR have been associated with different brain activity (Bosch et al., 2013). Taking all this evidence together, IR and DR may be based on dissociable neural substrates, or at least separate neural circuits.

However, no studies about neural correlates of IR and DR in traditional list-learning tests in the elderly have yet been conducted. While neuropsychological tests have been widely used to evaluate brain function, the underlying substrates of these test results have rarely been explored. Furthermore, different memory measures of IR and DR have been treated as a whole to evaluate general episodic memory function, making it impossible to detect the variability inherent in individual memory indices (Ystad et al., 2010; Wolk et al., 2011). Therefore, the potential differences in neural mechanisms underlying IR and DR in the elderly population need to be investigated.

Brain activation in resting state is highly similar to the functional activity in a cognitive task. It is increasingly believed that intrinsic activations shape patterns of cognitive task activations and predict cognitive ability (Smith et al., 2009; Cole et al., 2016; Schultz and Cole, 2016). Thus, resting state fMRI is a promising tool to explore intrinsic brain activity and its relationship with cognitive processes, with extensive use of regional homogeneity (ReHo) and functional connectivity (FC) analyses. ReHo supposes that voxels within a functional brain area are more temporally synchronous when this area is involved in a specific condition (Zang et al., 2004). It is applied in the prediction of variations in cognitive performance in healthy people by detecting regional signal changes (Tian et al., 2012). In addition, significant abnormalities in spontaneous low-frequency fluctuations coherence have been observed in patients with AD. ReHo has been speculated to be a biomarker of disease progression in patients with AD and MCI (He et al., 2007; Zhang et al., 2012). FC evaluates the interregional connectivity between spatially remote brain regions. Functional interactions between distinct regions also play a crucial role in cognitive processing and, similarly, altered patterns of FC is a proposed biomarker of AD and MCI (for a review, see van den Heuvel and Pol, 2010).

Therefore, in this study, we investigated the underlying brain systems of different episodic memory capacity in a cohort of normal elderly people using ReHo and FC of resting state fMRI signals. On the basis of previous work, we predicted that, as different memory retrieval indices, IR and DR depend on different neural correlates, and that DR may be supported by intercortical connections in memory-related brain regions to a greater extent.

## Materials and Methods

### Participants

A total of 70 cognitively normal older adults from local communities were included in the study. The participants met the following inclusion criteria: (1) age ≥ 60 years, (2) a score ≥22 on the Montreal Cognitive Assessment—Beijing Version (MoCA) (Yu et al., 2012), (3) no neurological deficits or traumatic brain injury, and (4) free of dementia and MCI. Data from four subjects were rejected due to consecutive excessive movement during fMRI scanning, resulting in data from a final total of 66 subjects (mean age = 70.18 years, range: 60–80 years; 26 men and 40 women).

This study was approved by the Ethics Committee of the Institute of Psychology of the Chinese Academy of Sciences, and all subjects signed informed consent forms.

### Assessment of Episodic Memory

Episodic memory function was measured by the Auditory Verbal Learning Test (WHO/UCLA version, AVLT) (Maj et al., 1994). The AVLT consists of 15 nouns from five different categories read aloud by the examiner (with an interval of 1 s between words) for five consecutive trials (trials 1 to 5), and each trial is followed by a free-recall test. The order of word presentation remained fixed across trials. Subjects were asked to repeat as many of the words as possible. After a 20-min delay period, subjects were again required to recall the words (trial 6). The number of correct items was recorded as the score in each trial.

We used the number of words recalled at the first trial as a measure of IR (scores range from 0 to 15), the number of words recalled at the last trial as a measure of DR (scores range from 0 to 15), and the total recall (TR) in trials 1 to 6 (scores range from 0 to 90) as a measure of episodic memory capacity.

### Neuroimaging Data Acquisition

Functional images were acquired under resting-state conditions with a 3-T Siemens scanner (Erlangen, Germany) at Beijing Imaging Center for Brain Research. Participants were instructed to relax, keep their head still and eyes closed, and not think about anything systematically or fall asleep. Functional images were obtained using an echo planar imaging sequence with the following parameters: time repetition = 2000 ms; time echo = 30 ms; flip angle = 90°; field of view = 200 mm × 200 mm; 33 axial slices; slice thickness = 3.0 mm; gap = 0.6 mm; acquisition matrix = 64 × 64; in-plane resolution = 3.125 × 3.125 and 200 volumes. To aid the localization of functional data, a high-resolution, three-dimensional T1-weighted structural image was also acquired for each subject with the following parameters: 176 slices; resolution = 256 × 256; voxel size = 1 mm × 1 mm × 1 mm; time repetition = 1900 ms; time echo = 2.2 ms; flip angle = 9°.

### Data Preprocessing and Analysis

#### Preprocessing

Resting-state fMRI data preprocessing was performed using Statistical Parametric Mapping (SPM8^1^), and a toolbox for Data Processing & Analysis for Brain Imaging (DPABI^2^) version 2.3 (Yan et al., 2016).

The first 10 volumes of each functional time series were discarded from the analysis to allow for magnetization equilibrium and for the acclimatization of the subjects to the scanning environment. The remaining 190 volumes were corrected for intravolume acquisition time differences between slices and were also corrected for intervolume geometrical displacement due to head movement. The functional images were then normalized to the standard space of the Montreal Neurological Institute (MNI) and resampled to a voxel size of 3 mm × 3 mm × 3 mm, and spatially smoothed with a 4-mm full-width at half-maximum (FWHM) Gaussian kernel. After that, the linear trend of the time courses was removed and temporal band pass filtering (0.01–0.08 Hz) was carried out to reduce the effects of low frequency drifts and physiological high-frequency noise. Participants included in this study were restricted to head motion of less than 2.0 mm in any direction and 2.0° of angular motion during the resting state scan. In addition, the volume-level mean framewise displacement (FD) (Van Dijk et al., 2012) was calculated and used as an extra global covariate in the next statistical analysis to exclude confounding effects of head motion. Moreover, an age-appropriate MRI template [i.e., Mayo Clinic Adult Lifespan Template (MCALT)] created recently for the analysis of elderly populations (Christopher et al., 2017) was used to verify the reliability of our results; for this, we performed the prepossessing and space normalization again while matching data with the MCALT.

#### ReHo Calculation and ReHo-Behavior Analysis

Following Zang et al. (2004), a within-subject analysis was first performed using the ReHo approach. The ReHo value in the brain was measured using Kendall’s coefficient of concordance (KCC) (Kendall and Gibbons, 1990) between the time series of a given voxel and its nearest 26 neighbors. Specifically, we first calculated the KCC for each voxel across the whole brain to derive the ReHo map for each subject. In order to reduce the effect of individual variability, each ReHo map was then divided by the mean ReHo value of the whole brain.

To examine whether ReHo of spontaneous activity varies with episodic memory capacity, we conducted correlation analyses between the total score of the AVLT (TR) and voxel-wise ReHo with age, gender, and years of education as confounding covariates. In further studies, the regions showing a significant correlation were defined as regions of interest (ROIs).

Next, to find the difference between IR and DR on ReHo of brain areas supporting episodic memory capacity, the mean ReHo value in each ROI was extracted, and the correlations between the ReHo value in the ROIs and performance of the two memory measures were then investigated.

#### FC Calculation and Connectivity-Behavior Analysis

The seed regions were defined by generating a 6-mm radius in ROIs based on ReHo-TR correlation results. The time series of each voxel in each ROI were averaged first. FC was then calculated as the correlation coefficients between the averaged time course of these ROIs. A Fisher’s r-to-z transform was then performed to convert the correlation coefficients to z values in order to improve the normality of these correlation coefficients.

We then examined whether or not any specific connections within the ROIs were able to predict memory performance; we expected to find a difference in FC for IR and DR. Correlations analyses between FC values in the ROIs and performance of the two memory variables were thus performed. In addition, differential effects between two correlation values were tested with Hotelling’s *t*-tests in an online software package at http://comparingcorrelations.org/ (Diedenhofen and Musch, 2015).

### Statistical Analyses

Statistical analyses for ReHo and FC were performed in DPABI. Statistical analyses of data for ReHo-behavior correlations and FC-behavior correlations were performed using SPSS 21.0. A cluster threshold at α < 0.05 (AlphaSim-corrected) for multiple comparisons was considered significant for ReHo analyses and FC analyses. This correction entailed a primary threshold of *p* < 0.01 with an extent threshold of 18 voxels (486 mm^3^; cluster connection radius *r* = 5 mm; the ROI mask and a resolution of 3 mm^3^). All the correlations analyses were calculated within a primary SPM 61^∗^ 73^∗^ 61 gray mask.

## Results

### Demographic and Behavioral Results

The characteristics and neuropsychological results of participants are shown in **Table 1**. MoCA scores ranged from 22 to 30 and AVLT total scores ranged from 31 to 86.

**Table 1:** Demographic characteristics and neuropsychological results (mean ± standard deviations).

Characteristics	Participants (*N* = 66)
Gender (female/male)	40/26
Age, years	70.18 ± 5.97
Education, years	13.79 ± 3.33
MoCA	27.02 ± 2.02
AVLT-TR	66.38 ± 11.99
AVLT-IR	7.11 ± 2.39
AVLT-DR	12.26 ± 3.00


*MoCA, Montreal Cognitive Assessment; AVLT, auditory verbal learning test; TR, total recall; IR, immediate recall; DR, delayed recall.*

### Correlations between the TR and ReHo of Spontaneous Activity in the Whole Brain (Gray Matter)

**FIGURE 1:**
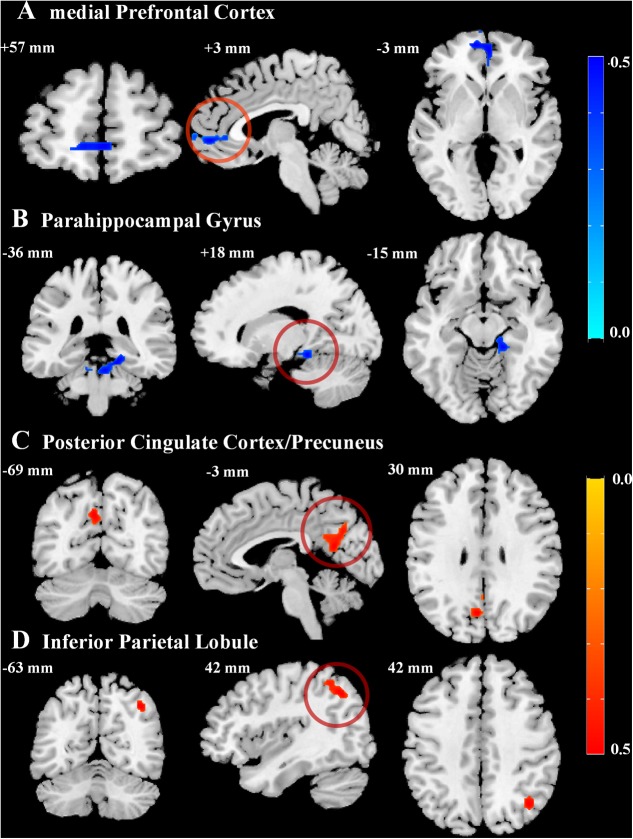
Correlations between episodic memory performance and regional homogeneity values. Statistical maps for the correlation between the total scores of Auditory Verbal Learning Test and regional homogeneity in critical regions of the default mode network (DMN), including the **(A)** bilateral medial prefrontal cortex, **(B)** right parahippocampal gyrus, **(C)** left posterior cingulate cortex/precuneus, and **(D)** right inferior parietal lobule (AlphaSim corrected cluster of *p* < 0.01). Negative (blue) and positive (red) correlation values are indicated by the bars on the right.

Voxel-wise correlation analyses showed that resting-state ReHo values correlated with AVLT-TR scores in a number of brain regions, most of which were located within the default mode network (DMN) (**Table 2**). The ReHo maps displayed very similar spatial patterns to the DMN proposed by a PET study by Raichle et al. (2001). Specifically, the ReHo values positively correlated with AVLT-TR scores in the posterior cingulate cortex/precuneus (PCC/PCu) and inferior parietal lobule (IPL), while negative correlations between the ReHo values and AVLT-TR scores were found in the medial prefrontal cortex (mPFC) and parahippocampal gyri (PHG) (**Figure 1**). In addition, using the data prepossessed with the MCALT, we replicated our results by finding significant correlations between the ReHo values and AVLT-TR scores within the critical DMN regions (i.e., the PCC/PCu, the IPL, and the PHG, all *p* < 0.01), except the mPFC. Therefore, the reliability of the main findings was confirmed with different templates.

**Table 2:** Regions showing significant correlations between the total score of Auditory verbal learning test and ReHo values.

Regions	L/R	BA	Peak MNI coordinates			*r*	Cluster size (voxels)
					
			*x*	*y*	*z*		
STG	L	38	-39	15	-24	-0.456^∗∗^	35
ITG	L	20	-57	-6	-36	-0.436^∗∗^	31
Cerebellum	L	18,19,37	-12	-63	-24	0.517^∗∗^	117
Cerebellum	R	18,19,37	21	-63	-18	0.398^∗∗^	61
SMG	L	21,22	-54	-54	24	0.417^∗∗^	26
PHG	R	30	18	-36	-15	-0.459^∗∗^	62
mPFC	L/R	10	-9	60	-3	-0.403^∗∗^	35
PCC/PCu	L	17,23	-3	-60	15	0.453^∗∗^	86
IPL	R	39,40	42	-63	42	0.395^∗∗^	32


*MNI, Montreal Neurological Institute; STG, superior temporal gyrus; ITG, inferior temporal gyrus; SMG, supramarginal gyrus; PHG, parahippocampal gyrus; MTG, middle temporal gyrus; mPFC, medial prefrontal cortex; PCC/PCu, posterior cingulate cortex/precuneus; IPL, inferior parietal lobule; L, left hemisphere; R, right hemisphere. ^∗∗^*p* < 0.001.*

### Immediate Recall, Delayed Recall, and ReHo of Spontaneous Activity within the DMN

**FIGURE 2:**
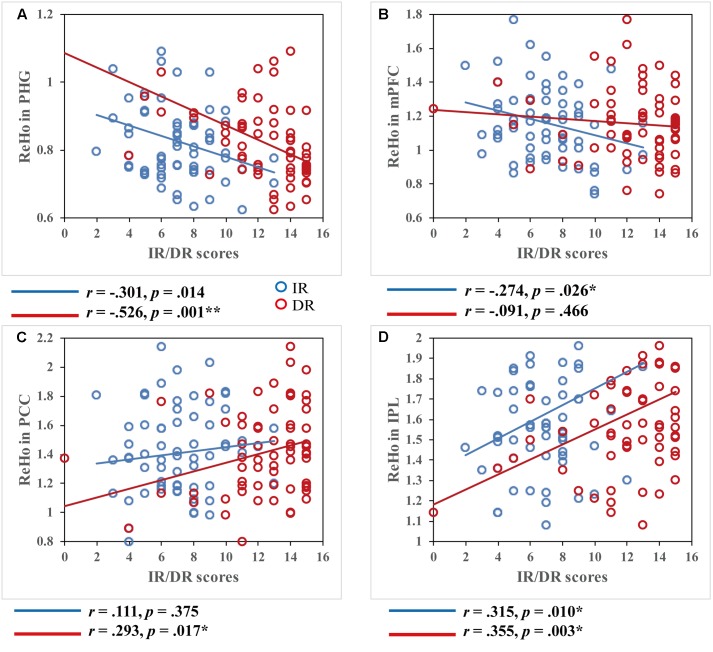
Correlations between regional homogeneity values and immediate recall (IR)/delayed recall (DR) scores. Both IR and DR scores were significantly correlated within most of the DMN regions, *p* < 0.05. Scatter plots display the relationship between regional homogeneity of the critical regions in the DMN, including the **(A)** bilateral medial prefrontal cortex, **(B)** right parahippocampal gyrus, **(C)** left posterior cingulate cortex/precuneus, and **(D)** right inferior parietal lobule and IR/DR scores, with age, gender, and education as covariates. Each dot represents data from one participant.

Further, we assessed the different contribution of regional coherence for spontaneous brain activity in the DMN to IR and DR. Based on the above results, the critical areas of the DMN [whose ReHo values showed a significant correlation with episodic memory performance (AVLT-TR)], including the PHG, mPFC, PCC/PCu, and IPL, were defined as ROIs. After extracting the mean ReHo value in each ROI, correlations between the ReHo measures of four ROIs, and IR and DR scores, respectively, were calculated. Significant correlations between ReHo values and memory were observed. As shown in **Figure 2**, IR score was positively correlated with ReHo values in the IPL (*r* = 0.315, *p* = 0.010) and negatively correlated with ReHo values in the PHG (*r* = -0.301, *p* = 0.014) and MFG (*r* = -0.274, *p* = 0.026). ReHo in two regions showed a significant positive correlation with DR scores, including the PCC/PCu (*r* = 0.293, *p* = 0.017) and IPL (*r* = 0.355, *p* = 0.003), while negative correlations were found within the PHG (*r* = -0.526, *p* < 0.001).

### Immediate Recall, Delayed Recall, and Functional Connectivity in the DMN

**FIGURE 3:**
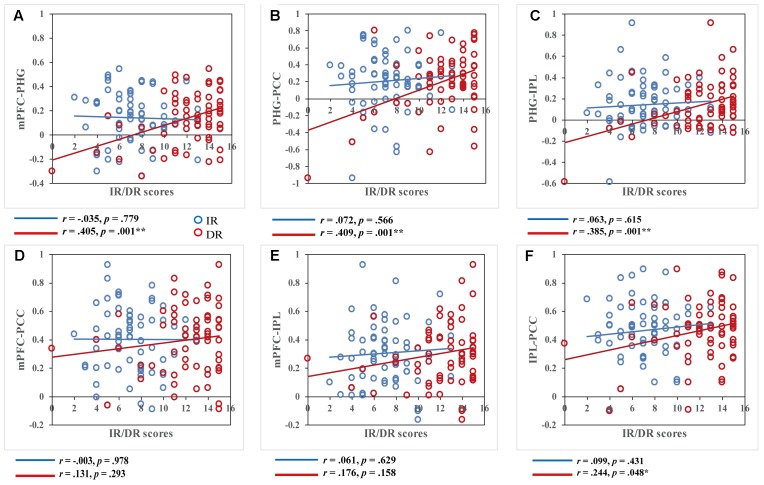
Correlations between functional connectivity values and immediate recall (IR)/delayed recall (DR) scores. DR scores were significantly correlated with functional connectivity within key regions of the DMN, while IR scores showed no correlation within these regions, *p* < 0.05. Scatter plots display the relationship between functional connectivity within the DMN, including functional connectivity of the **(A)** bilateral medial prefrontal cortex and right parahippocampal gyrus, **(B)** right parahippocampal gyrus and left posterior cingulate cortex/precuneus, **(C)** right parahippocampal gyrus and right inferior parietal lobule, **(D)** bilateral medial prefrontal cortex and left posterior cingulate cortex/precuneus, **(E)** bilateral medial prefrontal cortex and right inferior parietal lobule, and **(F)** right inferior parietal lobule and left posterior cingulate cortex/precuneus and IR/DR scores, with age, gender, and education as covariates. Each dot represents data from one participant.

We further examined the relationship between the remote FC and the two memory variables. All nine brain regions whose ReHo values showed a significant correlation with AVLT-TR were defined as the ROIs. After calculating a 9x9 correlation matrix between the time course of ROIs and the time series of all other ROIs and extracting the mean FC value of each ROI, we conducted correlation analyses between FC values in the ROIs and memory performance. No significant correlations between FC and IR scores or between FC and DR scores in brain regions not belonging to the DMN (after Bonferroni correction) were found. However, when focusing on regions located in the DMN, i.e., the PHG, mPFC, PCC/PCu, and IPL, the results were interesting. Although there were no significant correlations between FC values and IR scores, significant correlations were found between FC within the DMN and DR scores (**Figure 3**). More specifically, DR scores were significantly associated with PHG-MFG connectivity (*r* = 0.405, *p* = 0.001), PHG-PCC connectivity (*r* = 0.409, *p* = 0.001), and PHG-IPL connectivity (*r* = 0.385, *p* = 0.001). In addition, a significant correlation was found between DR scores and PCC-IPL connectivity (*r* = 0.244, *p* = 0.048) without Bonferroni correction. To test whether two correlation values of IR and DR scores were significantly different in these connectivity pairs, Hotelling’s *t*-tests were conducted. The results showed significant difference regarding the relationship of IR and DR scores and connectivity strength, in the PHG-MFG connectivity (*t* = -3.5425, *p* < 0.001), the PHG-PCC connectivity (*t* = -2.6613, *p* = 0.009) and the PHG-IPL connectivity (*t* = -2.5139, *p* = 0.015). It further supported the findings of correlations analyses.

## Discussion

We investigated the neural correlates of different verbal episodic memory indices, as defined by the AVLT, in a cohort of 66 healthy elderly participants. Using the ReHo method and FC analysis, we found that the ReHo of primary regions related to age-related decline of episodic memory capacity were all located within the DMN. Specifically, AVLT-TR was negatively correlated with regional coherence of local activity in the PHG and MFG, and positively correlated with that in the PCC and IPL. Furthermore, we demonstrated that IR and DR relied differentially on the DMN. ReHo of spontaneous activity in the DMN could significantly account for the variations in IR, while both ReHo and FC within DMN could account for variations in DR.

To our knowledge, this is the first study to report that ReHo of the DMN nodes is related to episodic memory in healthy older adults. The DMN is a particular brain system that shows greater activity during the resting state than during goal-oriented or attention-demanding tasks (Jeong et al., 2015). It contains a set of functionally connected brain regions, including the mPFC, PCC/PCu, IPL, lateral temporal cortex, and hippocampal formation (Raichle et al., 2001; Fox and Raichle, 2007; Buckner et al., 2008; Raichle, 2015). Multiple lines of research using resting-state FC analysis have revealed the contribution of the DMN to episodic memory, and abnormality in DMN connections has been suggested to be one basis for memory impairment in patients with neurodegenerative diseases. For example, greater functional connections within the DMN (e.g., MPF-LIP connectivity) (He et al., 2012), and between the DMN and the medial temporal lobe system (e.g., PCC-hippocampus connectivity) (McCormick et al., 2013), have been found to indicate better memory performance. Several resting state fMRI studies have shown that disrupted functional connections of the DMN is related to poor episodic memory performance (for a review, see Dickerson and Sperling, 2009), especially in patients with MCI and various stages of AD (Greicius et al., 2004; Mevel et al., 2011; Binnewijzend et al., 2012). Together, this previous work indicates that the DMN subserves episodic memory processing. In concordance with this idea, we found an association between memory function and ReHo of spontaneous activity in primary DMN brain areas in memory-unimpaired older adults. Furthermore, our findings extend previous work showing that, in addition to resting-state FC, ReHo in the crucial DMN nodes also predicts memory performance. Resting-state FC reflects inter-regional synchronization of time courses, whereas ReHo reflects intra-regional synchronization of time courses, which may be related to regional metabolic homogeneity (He et al., 2007).

An interesting finding in the present study was the different pattern of correlations between ReHo values in the DMN and memory function in normal older adults. In particular, AVLT-TR scores were positively correlated with regional coherence in the PCC/PCu and IPL, and negatively correlated with that in the PHG and MFG. Previous studies have also produced mixed results. For instance, He et al. (2007) found significant negative correlations between ReHo in the mPFC and Mini-Mental State Exam scores in patients with AD, but significant positive correlations between ReHo in the PCC/PCu and Mini-Mental State Exam scores. One explanation for these varied results is a functional dissociation of DMN components. Recently, it has been proposed that the DMN is not homogenous, as it has previously been considered (Uddin et al., 2009; Andrews-Hanna et al., 2010; Leech et al., 2011; Damoiseaux et al., 2012; Bado et al., 2014). Although the exact function and anatomy of distinct functional components is still unknown (e.g., anterior vs. posterior or ventral vs. dorsal), the notion of functional heterogeneity in the DMN and its dissociable roles during memory formation has been widely supported. For example, Maillet and Rajah (2014) found that activation in the left mPFC predicted encoding success; however, activation in the PCC was greater during unsuccessful encoding, which may reflect task-unrelated thoughts. Similarly, in another study, the PCC/precuneus and anterior DMN node in the mPFC also showed functional heterogeneity, with the former regions significantly activating during memory retrieval and the latter regions strongly deactivating (Sestieri et al., 2011). In line with these studies, our results showed that the mPFC and PCC/PCu played dissociable roles in memory formation in the healthy elderly adults, leading to different correlations with memory performance.

As another major discovery, our results revealed the discrepant neural circuits within the identical brain network between temporary memory store and the stores for long-term retention in older adults. Correlation analysis between different memory measures and resting state brain activity of the DMN demonstrated that age-related variety of immediate and delayed memory abilities relied on partially dissociable neural underpinnings. IR was related to the regional coherence of spontaneous activity in the DMN, and DR depended on both regional fluctuations and interregional integration within the DMN. Although the DMN is engaged in episodic memory processing, its different role in IR and DR in older adults was previously unclear. As far as we know, this is the first study to show that the DMN plays different roles in memory across different retention intervals in older adults.

Delayed recall may reflect memory consolidation in the elderly, which would explain its correlation with interregional connections within the DMN in the present study. On the one hand, there is gradual reorganization within long-term memory storage as time passes after learning. As a result, DR comprises memory consolidation. Our findings parallel the idea that IR trials may measure attention and working memory to a greater degree than DR trials, which predominantly tap retention, consolidation, and delayed retrieval effects (Shankle et al., 2005; Rabin et al., 2009). On the other hand, cortico-cortical pathways and hippocampal-neocortical interactions are generally considered as the neural correlates of consolidation, whereby consolidation relies on connections of brain regions. Meanwhile, patterns of neural activity when performing a task can be reproduced in resting state (Foster and Wilson, 2006; Albert et al., 2009), which is devoted to the reprocessing of past experiences. Miall and Robertson (2006) proposed that the DMN may support the off-line processing and consolidation of memories. Taken together, our findings that the DMN FC positively predicted DR performance provides further evidence for the role of the DMN in memory consolidation.

In addition, we also observed that ReHo of other regions were associated with episodic memory performance, mainly including the temporal cortex, the supramarginal gyrus (SMG), and bilateral cerebellum. The superior temporal cortex contains the primary auditory cortex and plays a crucial role in speech process and auditory information process (Binder et al., 2000; Zatorre et al., 2002; Leff et al., 2009; Rauschecker and Scott, 2009). Furthermore, the SMG is related to linguistic functions, such as phonological processing in the phonological-articulatory loop (Dehaene-Lambertz et al., 2005; Rauschecker and Scott, 2009). Therefore, it is reasonable to have found involvement of the temporal cortex and SMG because we used an auditory memory test. In addition, our results are consistent with the recent findings of cerebellum’s cognitive role. It is increasing recognized that the cerebellum is engaged in multiple domains of higher-order cognitive functions and emotional control (Schmahmann and Caplan, 2006), beyond the motor domain. Neuroimaging and neuropsychological evidence has shown that the cerebellum contributes to episodic memory processes, and patients with cerebellar lesions have been reported to suffer from episodic memory deficits (Gottwald et al., 2004; Mathiak et al., 2004; Weis et al., 2004). Thus, in the future, we may investigate the neural correlates of episodic memory in view of brain networks, combining the cerebellar network and the DMN.

Our study has some limitations that should be noted. First, we only recruited elderly adults with normal cognition, and our results cannot, therefore, be generalized to other populations. Future studies should recruit individuals with different cognitive statuses (i.e., patients with MCI or AD) to evaluate the changing trajectory of the roles of the DMN in episodic memory with aging and cognitive deterioration. Second, we did not consider the relationship between resting-state activity and task-related activity, though the latter could further support our results. For example, in a PET study, a significant correlation was found between the DR score and metabolism in the PCC (Brugnolo et al., 2014). A further study is required to combine the data during task and resting state and gather more conclusive evidence about the different contributions of the DMN to IR and DR. Third, we did not employ a younger control group in the present study. Thus, we cannot determine the effect of aging on the relationship of spontaneous activity in the DMN and recall performance. Further studies are required to investigate whether our observations reflect a normal phenomenon or an age-related alteration.

In summary, we explored episodic memory capacity of the healthy elderly adults using ReHo analysis of resting state fMRI data, providing vital insights into our understanding of brain activity of regional coherence and its cognitive implications. Our study indicates that ReHo, a feasible and easily applied approach, can be employed to characterize the neural correlations of traditional list-learning test results. Furthermore, the neural mechanism of memory consolidation has received increasing attention, and our findings reveal the possible relationship between the DMN and memory consolidation during wake for the first time. We also demonstrated that DMN activity measured with ReHo analysis can be used as a way to identify biomarkers for memory decline and neurodegenerative disease.

## Author Contributions

LH conceived the idea, designed the study, analyzed and interpreted data, and drafted the manuscript. RL, PW, and ZZ assisted the analysis and interpretation of data. JL participated in the writing and revision of the manuscript.

## Conflict of Interest Statement

The authors declare that the research was conducted in the absence of any commercial or financial relationships that could be construed as a potential conflict of interest.

## Footnotes

1 http://www.fil.ion.ucl.ac.uk/spm

2 http://rfmri.org
